# Expanding recruitment to medical school: the motivations for and perceived barriers to entry of non-science entrants to a graduate-entry medical course: a qualitative study

**DOI:** 10.1186/s12909-025-08193-5

**Published:** 2025-12-18

**Authors:** Jessica Sinyor, Lindsay Muscroft

**Affiliations:** University of Warwick, Coventry, United Kingdom

**Keywords:** Admissions, Graduate-entry medicine, Non-science, application barriers

## Abstract

**Background:**

Entering medical school as a graduate is an increasingly well-worn route to becoming a doctor in the UK. Recent expansion in medical school places and four-year graduate-entry (GEM) programmes will likely further increase the number of medical students with a previous degree. A growing number of GEM courses accept graduates with an undergraduate degree in a ‘non-science’ subject. However, the pre-entry and application experiences of these entrants to GEM degrees in the UK remain under-explored. In the context of expanding medical school places to applicants with increasingly diverse educational backgrounds, we investigated this cohort’s motivations for studying medicine and perceived barriers to entry into medical school.

**Methods:**

12 semi-structured interviews were conducted with students on the GEM MBChB programme at Warwick Medical School (WMS). We applied descriptive thematic analysis to the data, coding verbatim transcripts to generate themes.

**Results:**

Overarching themes for motivations were: educational, professional and personal factors. These were divided into sub-themes: educational factors were categorised as occurring at school, university or postgraduation. Professional factors included the transferability of existing skills and experience, and the perceived job satisfaction, career stability and progression of a career in medicine, which participants contrasted with previous professional experiences. Personal factors included changing direction during the COVID-19 pandemic, experiences as (a friend/relative of) a patient and influence from others. Participants reported several perceived barriers to entry into medical school: demanding entrance tests and eligibility requirements, lack of awareness about programmes accepting non-science graduates, academic anxieties, and a fear of falling behind in ‘life stages’.

**Conclusions:**

Additional obstacles to entry into medical school faced by non-science graduates undermine their potential as future doctors. Reviewing entrance requirements and distributing recruitment material to non-science graduates could dismantle some of these barriers.

## Background

Four-year graduate-entry medicine programmes, aiming to accelerate the production of doctors and broaden access to a career in medicine, represent an increasingly well-worn route to becoming a doctor in the UK [1–5]. The majority of these graduate-entry courses further contribute to efforts to diversify the medical profession by accepting “non-science” graduates with a previous degree in any subject, including Warwick Medical School (WMS) which first welcomed arts, social sciences and humanities graduates in 2013 (BMJ [6]). Evidence shows no significant difference in academic attainment at medical school between non-science graduates and science graduates, challenging the long-standing assumption that an undergraduate degree in science is better preparation for studying medicine than a non-science degree [2, 7, 8].

Recent and planned expansion in medical school places relies on growth in traditional undergraduate training and graduate-entry programmes [9, 10]. For the latter two routes into medicine, non-science graduates represent a pool of largely untapped potential. It has long been accepted that medicine is both an art and a science; the humanistic perspective inherent to the study of the arts and humanities may be particularly relevant to the person-centred approach called for by the GMC [7, 11–15]. Indeed, pre-medical education in non-science subjects is associated with enhanced interpersonal skills, and non-science graduates may bring social, ethical, psychological, communication and critical thinking skills, and a welcome diversity of thought to medicine [16–18]. To fill newly expanded places, medical schools could, therefore, consider how best to attract non-science graduates. Gaining insight into the motivations of these aspiring doctors – and barriers they perceive to entry into medical school – will support their recruitment.

Researchers have long sought to identify the factors that motivate students to pursue a career in medicine, however, the literature has largely overlooked graduate entrants’ motivations, which may differ from those of traditional undergraduate medical students [19–23]. Because of this scholarly gap, the literature has mostly failed to explore if previous degree subject (science or non-science) influences reasons for studying medicine.

While a number of qualitative investigations have illuminated the experience of studying medicine as a non-science graduate entrant, little is known about the application experiences of these students. Among non-science applicants to medicine in Canada, self-reported barriers to entry included admissions test geared toward those with a background in science [17]. However, against a background of expanding medical school places and efforts to diversify the medical pipeline in the UK, non-science entrants’ motivations to study medicine and perceived barriers to entry to medical school merit deeper study, both subjects being key to recruitment of this cohort.

## Methods

### Participants

12 students were recruited from the four cohorts of the MBChB programme at WMS, a graduate-entry course in the West Midlands with 757 students enrolled. The interviews were coded in order from one to twelve and by the tenth interview, no new themes were identified and it was therefore agreed that data saturation had been achieved. The 12 participants represented 10.5% of non-science entrants to the MBChB programme at WMS from 2020 to 2023, recruited from cohorts who had applied pre- and post- the COVID-19 pandemic, allowing the researchers to identify whether the pandemic influenced motivations [24]. Students were invited to participate via an online noticeboard accessible to all cohorts and the social media page of a peer-led non-science teaching group. Students with degrees in the arts, humanities or social sciences were eligible to participate [25].

### Interviews

**Fig. 1:**
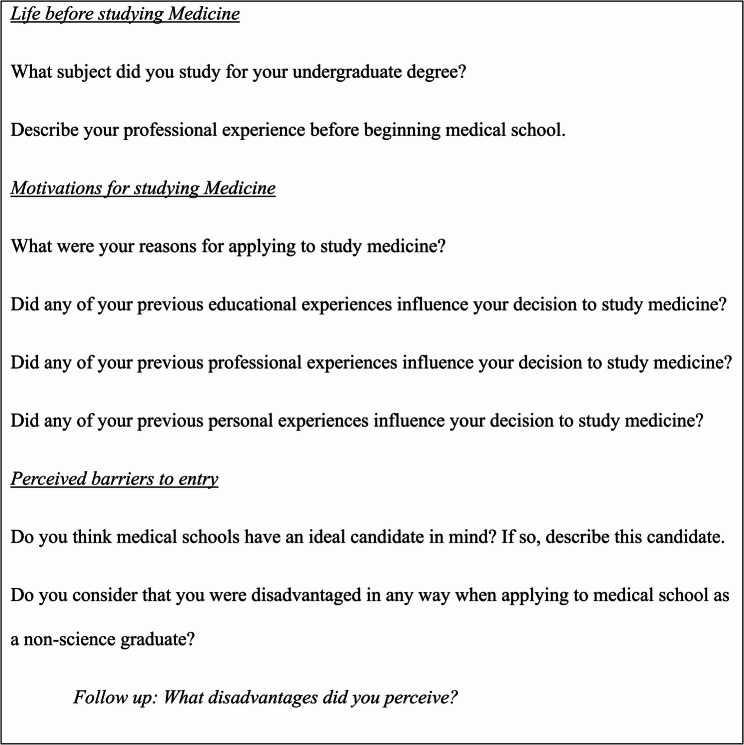
Schedule for semi-structured interviews

Previous studies of students’ motivations for studying medicine have employed surveys [19, 23]. However, as non-science entrants’ motivations are largely unexamined, semi-structured one-to-one interviews were used to enable a broad exploration of possible motivations. The pre-defined interview schedule (Fig. 1) was informed by Rapport et al.’s questions on the impact of participants’ past degree and experiences on current studies [26].

As the primary researcher [JS] is a non-science graduate entrant to the MBChB course at WMS, there was potential for bias towards answers that reflected their own experience. JS regularly reflected on their own perspective and discussed the interview process with the second researcher [LM], a member of the WMS faculty and a science graduate, to mitigate this potential. JS particularly reflected on how their application to medical school had given them a personal perspective on barriers to entry for non-science graduates. To avoid their own experience influencing participants’ responses, JS excluded any mention of their educational background from interviews. In addition, using an interview schedule produced by both JS and LM diminished the likelihood that participants would be asked leading questions and provided a structure that supported a neutral response from the researcher to participants’ answers. Ethical approval was obtained from the University of Warwick Biomedical & Scientific Research Ethics Committee (BSREC).

All interviews took place remotely via Microsoft Teams and were approximately 25–30 min in length. The audio and visuals were recorded for the purposes of checking Teams’ auto-transcripts for accuracy.

Using NVivo (Lumivero: Colorado) software, the two researchers [JS and LM] analysed the data using Braun and Clarke’s six step model for thematic analysis. Data was coded separately by the two researchers who independently reflected on the results and then discussed, further reducing the potential for bias.

## Results

### Participants

The 12 participants were drawn from all four cohorts at WMS, with two first year students, five second years, four third years and one final year student. There was a diverse group of participants, ranging in age from 23 to 32 years old with a mean age of 27. Eight of the participants identified as female, three as non-binary and one as male. Nine participants declared their ethnicity as White, two declared Mixed/Multiple Ethnic Background and one identified as Asian/Asian British. Five participants had completed degrees in the arts, four in the humanities (including foreign languages), and three in the social sciences.

### Themes

The themes were divided into the motivations for studying medicine of non-science entrants to a graduate-entry medical degree (RQ1) and perceived barriers to entry (RQ2). Seven themes were generated, alongside a number of sub-themes (Table 1).

**Table 1:** Thematic headings and sub-themes used in the analysis, with verbatim illustrative quotations

RQ1: What motivates non-science entrants to a graduate-entry medical degree to study medicine?		
Theme	Sub-theme	Illustrative quotation
Educational factors	At school	I had a really good biology teacher […] that made me consider maybe I’d like to work in that area. (Participant 3) I told a teacher that I wanted to be a doctor and […] he basically said that he didn’t think I had it in me to do it. (Participant 8)
	At university	The arts in general I think are about understanding people from a creative perspective and I think medicine is about understanding people from a biological and a psychological perspective […] At the root of it is just trying to understand people and so I do think that my degree and my background has helped me with that element. (Participant 10)
	Post-graduation	Coming from profession where I was a little bit bored […] back into an environment where you could learn has actually also been part of the appeal. (Participant 4) I wanted to do something that was like studying, but had a clear end goal in work. (Participant 7)
Professional factors	Perceived job satisfaction	I also was really needing some kind of direction and some kind of challenge, but also something that was going to be long term and not something that I was going to be challenged by and find exciting short term and then be like, OK what’s next? (Participant 2) I did see in medicine an opportunity to use my intellect in a variety of ways which weren’t all about myself. (Participant 6)
	Perceived career stability and progression	The crux of it with any arts or entertainment is that it’s really unstable. There’s no guaranteed career or career progression […] I knew that going into medicine was almost like a guaranteed progression and that was very appealing. (Participant 10)
	Transferability of existing skills and experience	Obviously, when you’re doing music with them, it’s therapeutic. It has therapeutic value in a different way to the sort of therapeutic value of performing medicine. (Participant 3) I’m also quite good at talking to lots of different types of people, because the whole thing about acting is you need to be able to empathise to a point where you can literally feel what they’re feeling. (Participant 12)
Personal factors	Changing direction during the COVID-19 pandemic	That was about the same time as the pandemic […] I thought I really want to be out there helping, doing something and I was kind of stuck inside pretending to be a giraffe on Zoom and it all just felt a bit farcical. (Participant 12) Over lockdown and over 2020 I had a lot of time to think […] I guess something just kind of flipped in my brain and I was like it’s not too late. (Participant 2) I was kind of in a place where I was re-evaluating life on a number of levels. (Participant 4)
	Experiences as a patient, or friend/family member of a patient	I’ve had, personally, some bad experiences with doctors. And I think in a way, it kind of made me feel empowered to become one myself so that I can help drive some change. (Participant 10) My mum getting cancer was the main thing that made me think about becoming a doctor just because, when you’re going through that kind of experience, you’re exposed to healthcare and the good and bad things about it. (Participant 9)
RQ2: What are non-science graduates’ perceived barriers to entry into medical school?		
Theme	Sub-theme	Illustrative quote
Application requirements	Clinical experience	If you’re working a full-time job and then needing to find what, 70 h [of clinical experience] I think that can be a barrier. (Participant 4)
	Eligibility to apply	Without a science background, obviously I was limited in the universities I could apply to. (Participant 1) I was thinking about applying to [my undergraduate university to study medicine] and I asked the tutor about it. And it was like, “Oh, how much physics did you do? But you’d be so great. We’d love to have for you. But obviously, these are requirements. We can’t.” I’m like, but why? You can do an astronomy degree [and be eligible to apply for medicine]. So I think it’s just the arbitrariness of it, I think is frustrating. (Participant 7)
	Entrance tests	The GAMSAT is a scary exam for someone who hasn’t done any science since GCSEs. (Participant 12) It’s quite hard to do well in the science aspects of the GAMSAT if you’ve not done science before. It’s quite a steep learning curve. (Participant 5)
Lack of awareness of programmes accepting non-science graduate applicants		I didn’t really know, to be honest, that graduate courses existed. (Participant 11) I thought I was going to have to go back and do A Levels again. (Participant 12) There was a girl who was two years ahead of me who did a politics degree, who then went and studied graduate entry medicine […] that kind of triggered it for me. (Participant 3)
Life stage		By the time that you’re doing a postgrad, there’s also just the idea of, OK, age, money and lots of things like that. Can I fund myself for this degree? Am I on track with my life if I do another degree? (Participant 9)
Academic anxieties		I’m still getting definite imposter syndrome […] I did wonder about whether I’d have the brain capacity to learn everything. (Participant 1) You kind of think, well, why would they let me in with my music degree? (Participant 10)

RQ1: What motivates non-science entrants to a graduate-entry medical degree to study medicine?

### Educational factors

Experiences of studying science at school proved both a motivator and a de-motivator for choosing medicine. Some participants described inspirational teachers who gave them an enduring enthusiasm for science, which later consolidated their decision to study medicine. For others, discouraging teachers, the impression that aspiring doctors needed to be “*top tier smart*” (P1) and needing to decide between arts and sciences at A-Level meant that they decided against medicine while at school.

Reflecting on how their time at university motivated them, several participants drew connections between the analytical and humanistic approaches prized by the arts and humanities and their present studies, observing that medicine may be a more natural next step than might be assumed. “*The root of [both] is just trying to understand people*,” (P10) said one student, while another described the “*therapeutic value*” (P3) shared by their previous degree in music and their medical studies.

An interest in post-graduate education with the degree of structure and purpose attached to studying medicine was cited by participants as a strong motivator. “*I am much more able to achieve when I have something that’s laid out for me*,” (P2) noted one interviewee, while another was anxious that any further study should have “*a clear end goal*” (P7) or vocation, in contrast to their previous studies.

### Professional factors

Interviewees were motivated by the job satisfaction they perceived as inherent to a career in medicine. Intellectual challenge, impactful work and the sense of direction medicine promised were contrasted with the participants’ previous careers, which some interviewees felt lacked impact: “*I did see in medicine an opportunity to use my intellect in a variety of ways which weren’t all about myself*” (P6).

Perceived job stability and career progression were key factors that motivated participants to pursue medicine over existing careers. The jobs that interviewees sought after non-science degrees – in film production, events, academia, music and teaching – were described as poorly paid, lacking stability and opportunities to progress. This contrasted with their impression of a career in medicine, which they perceived as stable with a clear structure for progression.

Participants also described how work gained in other sectors clarified aspects of work they enjoyed. Working in a school, in events and in a choir helped interviewees to realise that they enjoyed working with people, in a team, “*moving around*” (P7), in “*fast-paced*” (P2) environments. Reflecting on the more rewarding aspects of their jobs and realising that a career as a doctor “*tick[ed] all the boxes*” (P10) was a common path to applying to study medicine.

### Personal factors

Interviewees identified experience of healthcare as a patient or family member/friend of a patient as a motivator. Often these were “*bad experiences*” (P10), encouraging the student to enter medicine to “*drive change*” (P10). The COVID-19 pandemic also proved motivating in giving interviewees “*time to think*” (P2), allowing them to “*re-evaluate*” (P4) their career priorities. For some interviewees, the pandemic consolidated their desire to do something they found more meaningful than their present occupation or studies.

 RQ2: What are non-science graduates’ perceived barriers to entry into medical school?

### Application requirements

There was a consensus among interviewees that the number of universities to which they could apply as non-science graduates was a barrier to entry. While they did not feel disadvantaged once they reached interview stage, interviewees felt that the “*entry point*” (P12) was unfairly restricted by eligibility criteria or admissions tests. Requirements for A-Levels or degrees in science for many graduate medicine courses provoked dissatisfaction: “*I don’t think I necessarily couldn’t have thrived at those other universities*” (P3). One interviewee was frustrated by the perceived arbitrariness of any science degree as a prerequisite of entry for some courses, arguing that “*rigour*” is similarly characteristic of studies in the humanities: “*[Philosophy] is really scientific and rigorous*,* as are other humanities subjects*” (P7). Another noted that “*medicine is an enigma*,* such that nobody is ever really prepared*,* no matter what degree you come from*” (P3).

Interviewees were also united in their impression that the Graduate Medical School Admissions Test (GAMSAT), currently used by 12 universities in the UK, deters non-science graduate applicants from applying to more medical schools, thereby reducing their chances of making a successful application [27]. Only one of the interviewees had attempted the GAMSAT, with others describing the exam as “*really expensive*” (P10) at £271 and “*horrible*” (P3) [27]. Participants felt the scientific demands of the exam were unrealistic for graduates of non-science degrees. It should be noted that this finding may be an artefact of the sampling, given that all participants hailed from a university which required only the UCAT: this is explored in greater depth in the discussion.

Several interviewees were discouraged from applying by university-specific eligibility requirements for healthcare-related work experience, with WMS requiring applicants to complete 70 h [28]. Interviewees used family and friends to organise placements, which, one observed, is “*not something everyone would be able to do*” (P4).

### Lack of awareness of programmes accepting non-science graduate applicants

A lack of awareness that some graduate-entry medicine degrees accept non-science entrants proved a barrier to entry. There was frequent mention of not “*realis[ing] grad entry med was a thing*” (P10) or that they could study medicine without sitting further A-Levels. Some participants cited this as one reason that they had not applied to medical school sooner. There was a consequent reliance on informal networks: interviewees who had closer “*proximity to doctors*” (P6) mentioned these family and friends both as informers (about graduate-entry medicine) and influences on their own choices. Friends who had “*gone to study medicine […] from non-medical backgrounds*” (P2) were particularly instructive, modelling this less conventional route for interviewees and “*trigger[ing]*” (P3) their own interest.

### Life stage

Fears around being “*on track with […] life*” (P9), being “*too old*” (P2), reconciling family life with a long training pathway, and the financial implications of funding another degree were a barrier. While these worries are not unique to non-science graduates, having put time into an unrelated career added a layer of complexity to “*giving all that away*” (P4). Several interviewees had worked for upwards of five years in non-healthcare related fields and had “*invested so much*” (P6) in their jobs: starting a medical degree felt like sacrificing this investment.

### Academic anxieties

Several interviewees described concerns about the science requirements of studying medicine as a non-science graduate as a barrier. Faced with the competitiveness of entry into medical school, interviewees described “*imposter syndrome*” specifically related to their educational background: “*why would they let me in with my music degree?*” (P1, P10).

## Discussion

When compared to previous literature, our findings show that non-science graduates’ reasons for studying medicine differ from those given by aspiring undergraduate medics, for whom an interest in science is a significant motivating factor [19, 21, 22]. While the participants in the present study cite the appeal of intellectual challenge and continued learning, a specific interest in science did not come through in the data as a motivation. Status and salary, previously identified as motivating factors, were also not mentioned [19, 21, 22].

Non-science graduates show specific motivations for studying medicine, influenced by connections they found between the approaches of the arts/humanities and medicine and a desire to continue learning consolidated by undergraduate studies. Where there was overlap in motivations between aspiring undergraduate medical students and the present study’s participants, it was in personal experience of what Isik et al. [22] describe as “getting acquainted with the medical profession”. In-keeping with previous research, having medical professionals as friends or family encouraged participants to pursue medicine or personal experience as (family of) a patient, an experience likely common to most aspiring doctors, although perhaps particularly pertinent for graduate-entry medics by virtue of being older cohort with older parents/relatives [22].

Professional factors that motivate non-science graduates include their view of a doctor’s career as stable and satisfying compared to precarious jobs in sectors such as the arts, in contrast to previous studies in which graduate medics mourned “amazing jobs […] being foregone” [26]. From the perspective of non-science graduates specifically, the careers available to them compared ill-favourably with medicine in terms of stability, progression and job satisfaction. A need for professional fulfilment resonates with previous studies of social sciences and humanities graduates studying medicine [17]. However, the security and career progression these future doctors describe is threatened by a training bottleneck that may deter applications from graduates for whom stability was a key attraction [29–31].

Investigating non-science graduates’ motivations reveals that this is a diverse cohort whose reasons for studying medicine demonstrate analytical and reflective skills, and a commitment to continued learning. However, against the backdrop of fierce competition for medical school places, we found additional perceived barriers to entry. A lack of awareness about programmes accepting non-science entrants disadvantages those without existing contacts in healthcare, undermining the role of graduate-entry programmes in widening participation. This finding parallels a recent investigation into the influence of socioeconomic background on medical school choice which recommended that medical schools tailor application guidance to applicants from non-traditional backgrounds [32]. This could be as straightforward as university careers services disseminating information about graduate medicine as an option for arts, humanities and social sciences students. On the recruitment side, medical school admissions offices could feature profiles of successful non-science entrants in their marketing, showcasing ‘mentor’ figures in whom applicants can see themselves reflected. This could also tackle the imposter syndrome we found non-science graduates felt, which specifically related to their previous studies.

This study reveals that non-science graduates perceive several aspects of applying to medical school to be particularly challenging for them to tackle, including clinical experience requirements, eligibility criteria and entrance tests that deter those without a recent background in science from applying. Difficulties arranging clinical experience is, of course, not unique to non-science graduates. However, committing a significant amount of time to gaining experience, for example by volunteering in healthcare, may present an additional challenge if applying while working rather than studying, as was the case for several participants [33]. While acknowledging the additional burden this may place on some applicants, the authors agree that work experience remains a vital aspect of applying to medical school [34]. Clinical experience offers candidates the opportunity to confirm their commitment to medicine to both themselves and their prospective university, which is perhaps particularly important for applicants pivoting into an entirely new career.

Courses open only to graduates with a degree in any science, including non-biological sciences, provoked frustration. Some participants felt that this demonstrated that course providers could teach pre-clinical sciences without relevant existing knowledge of life sciences, in which case they felt that they should not be restricted from applying. However, it should be noted that the perception among participants that they could only apply to a handful of universities due to their lack of science A-Levels and undergraduate degree is increasingly inaccurate, with the majority of GEM programmes now open to this cohort.

Participants had, in fact, often truncated the list of universities to which they could apply by declining to consider courses requiring the GAMSAT. The GAMSAT was found to be so off-putting to study participants that applicants excluded themselves from consideration by universities whose entry criteria they otherwise fulfilled. As the participants had all completed the University Clinical Aptitude Test (UCAT) to apply to WMS, and had scored sufficiently highly to make competitive applications, there may be a degree of sampling bias in the finding that the GAMSAT presented an insurmountable challenge for some non-science applicants. Further research into the perspectives and application experiences of non-science students at medical schools requiring the GAMSAT is necessary before the authors could consider recommending that course providers collaborate to standardise entrance tests requirements. In addition, wider adoption of the UCAT, for example, could result in new barriers being erected: some demographic groups, including female applicants, perform less well in the assessment, problematising its wider deployment [35].

Other perceived barriers to entry were erected by the interviewees’ own perception of their likely success on the course. Allaying these fears may be as straightforward as including data that non-science graduates ultimately perform equally well as their science graduate counterparts in recruitment material [2, 7, 8]. Additionally, course providers who offer specialised teaching for non-science entrants should highlight this information to potential applicants.

### Limitations

Future studies should undertake a direct qualitative comparison between non-science and science graduate entrants’ motivations and perceived barriers to entry. The small sample size and gender demographics of the participants both represent limitations: while the number of female participants reflected those of medical students at WMS (64.6%), male students were under-represented, representing 8.3% of participants despite comprising 34.8% of the medical student body at WMS [24]. Nationally, 38.3% of accepted applicants to medicine in 2024 identified as male, while 61.1% identified as female [36]. Non-binary students were over-represented among participants, with only 0.64% of medical students at WMS identifying as non-binary or ‘other’, and 0.0062% of national applicants selecting ‘I prefer not to say’ or ‘I use another term’ for their UCAS application [36]. However, there were no themes explicitly related to gender in the answers given by the participants, suggesting that gender identity did not particularly impact results [24]. That participants all hailed from a single institution is a further limitation, although the length of the interviews and the range of the questions asked still afforded rich qualitative data. Finally, the data relied on participants recalling past feelings and events, so memory or subsequent processing of experiences may challenge the accuracy of the motivations and barriers as recollected by participants.

## Conclusions

As universities expand places on medical degrees to widen access to a career in medicine and meet the needs of the NHS, these findings give new insight into how course providers can better support non-science graduate applicants to reach medical school. In the drive to deliver person-centred care, the wisdom of recruiting from this cohort is based on the skillset fostered by an education in the arts, humanities and social sciences, including reflectiveness, communication skills, and an understanding of a person in their socio-economic and cultural context. This education arguably provides an equally robust foundation for studying medicine as the knowledge gained in the course of a science degree. As with previous research showing the absence of an attainment gap between non-science and science entrants to graduate-entry medical degrees, this study vindicates widening access to the medical profession and should prompt universities to reconsider entry criteria that preclude or particularly challenge applications from this cohort [2, 8]. However, soaring places at medical school must be matched by increased training places to ensure that doctors can progress into the fulfilling, secure careers elucidated by this study [29]. As the medical profession expands, dismantling obstacles to entry for graduates from different educational backgrounds will open medicine up to a richer variety of skills, experience and perspectives [37].

## Data Availability

The data that support the findings of this study, i.e. transcripts of the interviews with participants, are available on request from the corresponding author.

## Abbreviations

GEM: Graduate-entry medicine
MBChB: Bachelor of Medicine, Bachelor of Surgery
WMS: Warwick Medical S chool
GAMSAT: Graduate Medical School Admissions Test
UCAS: Universities and Colleges Admissions Service
UCAT: University Clinical Aptitude Test

## References

[1] Garrud P. Who applies and who gets admitted to UK graduate entry medicine? - An analysis of UK admission statistics. BMC Med Educ. 2011;11:71. 21943332 10.1186/1472-6920-11-71 PMC3196729

[2] Garrud P, McManus IC. Impact of accelerated, graduate-entry medicine courses: a comparison of profile, success, and specialty destination between graduate entrants to accelerated or standard medicine courses in UK. BMC Med Educ. 2018;18(1):250. 30400933 10.1186/s12909-018-1355-3 PMC6219209

[3] Manning G, Garrud P. Comparative attainment of 5-year undergraduate and 4-year graduate entry medical students moving into foundation training. BMC Med Educ. 2009;9:76. 20028543 10.1186/1472-6920-9-76 PMC2808300

[4] Price R, Wright SR. Comparisons of examination performance between ‘conventional’ and graduate entry programme students; the Newcastle experience. Med Teach. 2010;32(1):80–2. 20095780 10.3109/01421590903196961

[5] Shehmar M, Haldane T, Price-Forbes A, Macdougall C, Fraser I, Peterson S, Peile E. Comparing the performance of graduate-entry and school-leaver medical students. Med Educ. 2010;44(7):699–705. 20636589 10.1111/j.1365-2923.2010.03685.x

[6] BMJ Careers. Applying To Medical School: A Step by Step Guide BMJ Careers2022 [updated 11 August 2022. Available from: https://www.bmj.com/careers/article/applying-to-medical-school-a-step-by-step-guide

[7] Finucane P, Flannery D, McGrath D, Saunders J. Demographic attributes and knowledge acquisition among graduate-entry medical students. Med Teach. 2013;35(2):134–8. 23102104 10.3109/0142159X.2012.733833

[8] Aston-Mourney K, McLeod J, Rivera LR, McNeill BA, Baldi DL. Prior degree and academic performance in medical school: evidence for prioritising health students and moving away from a bio-medical science-focused entry stream. BMC Med Educ. 2022;22(1):700. 36195862 10.1186/s12909-022-03768-y PMC9533538

[9] 350 extra medical. school places allocated in NHS training boost [press release]. 2024.

[10] NHS England. NHS Long Term Workforce Plan. 2023.

[11] General Medical Council. Outcomes for Graduates. 2018 2018.

[12] Saunders J. The practice of clinical medicine as an Art and as a science. Med Humanit. 2000;26(1):18–22. 12484313 10.1136/mh.26.1.18

[13] Strange TJ, Castellanos MR. Medicine—Both a science (Care) and an Art (CARE). JAMA. 2024;331(16):1357–8. 38568598 10.1001/jama.2024.2508

[14] Francis G. Medicine: Art or science? Lancet. 2020;395(10217):24–5. 31982047 10.1016/S0140-6736(19)33145-9

[15] Herman J. Medicine: the science and the Art. Med Humanit. 2001;27(1):42–6. 23670550 10.1136/mh.27.1.42

[16] Mangione S, Chakraborti C, Staltari G, Harrison R, Tunkel AR, Liou KT, et al. Medical students’ exposure to the humanities correlates with positive personal qualities and reduced burnout: A Multi-Institutional U.S. Survey. J Gen Intern Med. 2018;33:628–34. 29380213 10.1007/s11606-017-4275-8 PMC5910341

[17] Lam JTH, Hanson MD, Martimianakis MAT. Exploring the socialization experiences of medical students from social science and humanities backgrounds. Acad Med. 2020;95(3):401–10. 31348068 10.1097/ACM.0000000000002901

[18] Stratton TD, Elam CL, McGrath MG. A Liberal arts education as Preparation for medical school: how is it valued? How do graduates perform? Acad Med. 2003;78(10 Suppl):S59–61. 14557097 10.1097/00001888-200310001-00019

[19] Crossley ML, Mubarik A. A comparative investigation of dental and medical student’s motivation towards career choice. Br Dent J. 2002;193(8):471–3. 12516673 10.1038/sj.bdj.4801599

[20] Vaglum P, Wiers-Jenssen J, Ekeberg O. Motivation for medical school: the relationship to gender and specialty preferences in a nationwide sample. Med Educ. 1999;33(4):236–42. 10336753 10.1046/j.1365-2923.1999.00293.x

[21] Holzer BM, Ramuz O, Minder CE, Zimmerli L. Motivation and personality factors of generation Z high school students aspiring to study human medicine. BMC Med Educ. 2022;22(1):31. 35016664 10.1186/s12909-021-03099-4 PMC8753872

[22] Wouters A, Isik U, Ter Wee MM, Croiset G, Kusurkar RA. Motivation and academic performance of medical students from ethnic minorities and majority: a comparative study. BMC Med Educ. 2017;17(1):233. 29183363 10.1186/s12909-017-1079-9 PMC5706443

[23] Pagnin D, De Queiroz V, De Oliveira Filho MA, Gonzalez NV, Salgado AE, Cordeiro e Oliveira B, et al. Burnout and career choice motivation in medical students. Med Teach. 2013;35(5):388–94. 23458255 10.3109/0142159X.2013.769673

[24] University of Warwick. (Obtained under the Freedom of Information Act, received 19 July 2024). 2024.

[25] Ellaway RH, Bates A, Girard S, Buitenhuis D, Lee K, Warton A, et al. Exploring the consequences of combining medical students with and without a background in biomedical sciences. Med Educ. 2014;48(7):674–86. 24909529 10.1111/medu.12496

[26] Rapport F, Jones GF, Favell S, Bailey J, Gray L, Manning A, et al. What influences student experience of graduate entry medicine? Qualitative findings from Swansea school of medicine. Med Teach. 2009;31(12):e580–5. 19995159 10.3109/01421590903193570

[27] Australian Council for Educational Research. GAMSAT: Australian Council for Educational Research. 2023 [Available from: https://gamsat.acer.org/university-admission/universities

[28] University of Warwick. Work experience: University of Warwick. 2023 [Available from: https://warwick.ac.uk/fac/sci/med/study/ugr/applying/entryreqs/workex/

[29] Dobson J. Training bottlenecks and unemployment—bad for doctors, patients, and the public purse. BMJ. 2025;389:r1204.

[30] Beard A, Gill B. Medical registrar bottleneck. Postgrad Med J. 2022;99(1171):370–1. 10.1136/pmj-2022-142099 37294719

[31] Oliver D. David oliver: bottlenecks in postgraduate medical training are an abject failure of medical leadership. BMJ. 2025;389:r1180. 40500136 10.1136/bmj.r1180

[32] Rees EL, Mattick K, Harrison D, Rich A, Woolf K. I’d have to fight for my life there’: a multicentre qualitative interview study of how socioeconomic background influences medical school choice. Med Educ Online. 2022;27(1):2118121. 36048126 10.1080/10872981.2022.2118121 PMC9448433

[33] McHarg J, Mattick K, Knight LV. Why people apply to medical school: implications for widening participation activities. Med Educ. 2007;41(8):815–21. 17661890 10.1111/j.1365-2923.2007.02798.x

[34] Council MS. Work experience 2025 [Available from: https://www.medschools.ac.uk/for-students/applying-to-medical-school/work-experience/

[35] Griffin B, Horton GL, Lampe L, Shulruf B, Hu W. The change from UMAT to UCAT for undergraduate medical school applicants: impact on selection outcomes. Med J Aust. 2021;214(2):84–9. 33258184 10.5694/mja2.50877

[36] UCAS. Undergraduate End of Cycle Data Resources 2024 2025 [Available from: https://www.ucas.com/data-and-analysis/undergraduate-statistics-and-reports/ucas-undergraduate-end-cycle-data-resources-2024

[37] British Medical Association. Medical staffing in England: a defining moment for doctors and patients. 2021.

